# Comorbid chronic diseases and their associations with quality of life among gynecological cancer survivors

**DOI:** 10.1186/s12889-015-2240-1

**Published:** 2015-09-25

**Authors:** Ji-Wei Wang, Li Sun, Jiang Li, Xiao-Huan Cong, Xue-Fen Chen, Zheng Tang, Dong-Hui Yu, Tian-Rui Zhang, Zheng-Nian Luo, Zheng-Ping Yuan, Jin-Ming Yu

**Affiliations:** School of public health and Key Laboratory of Public Health Safety, Fudan University, 30 Dong-An Road, Shanghai, 200032 China; Jonathan and Karin Fielding School of Public Health, University of California, 650 Charles Young Drive South, Los Angeles, CA 90095 USA; College of Clinical Medicine, Anhui Medical University, Hefei, Anhui Province China; Shanghai Health Education Association, 122 Shan-Xi-Nan Road, 200040 Shanghai, China; Shanghai Cancer Rehabilitation Club, 2795 Yang-Gao-Zhong Road, Shanghai, 200135 China

**Keywords:** Gynecological cancer survivors, Quality of life, Comorbid chronic diseases, Cancer care

## Abstract

**Background:**

Many gynecological cancer survivors (GCS) have comorbid chronic diseases (CCD). This study was to estimate the impacts of CCD on quality of life (QOL) in GCS.

**Methods:**

We collected cross-sectional self-reported survey data from 598 GCS between April and July 2013, in Shanghai, China. All the subjects were asked to complete a questionnaire containing the European Organization for Research and Treatment quality of life version 3 questionnaire (EORTC QLQ-C30) and questions on socio-demographic characteristics and CCD. In order to mitigate the bias caused by confounding factors, multiple linear models were employed to calculate adjusted means of QOL scores.

**Results:**

Approximately three-quarters of subjects reported at least one CCD. The highest overall prevalence of all CCD was found in endometrial cancer survivors. Subjects with CCD generally reported lower scores for most EORTC QLQ-C30 scales when compared to subjects without CCD, indicating poorer QOL, particularly for cardiovascular diseases, respiratory diseases, digestive diseases, and musculoskeletal disease.

**Conclusions:**

The CCD are common health problems among GCS. CCD have significantly negative influence on QOL, and GCS with CCD generally reported lower QOL scores. These findings suggested comprehensive cares for GCS.

## Background

Gynecologic cancers are the third most common female cancer, occurring in about 1 in 20 women worldwide [1], and are a major source of mortality and morbidity [2]. Due to population expansion and increasing proportion of the elderly population, the number of gynecological cancers in China is rapidly increasing [3]. With the exception of ovarian cancer, most gynecological cancer diagnoses are associated with good survival rates [4–6]. Thus, more and more Chinese women who will be long-term gynecologic cancer survivors (GCS) are added to the population each year [3].

Despite longer survival time, GCS may continuously live with conditions such as long-term and late physical, psychosocial and sexual effects of gynecological cancer and treatment [7, 8], as well as associated comorbid chronic diseases (CCD) that influence their survival and quality of life (QOL). Assessment of QOL to monitor and support long-term GCS for both medical and psychologic consequences of their cancer treatment and rehabilitation is imperative. A few studies to date have examined the impact of CCD on gynecologic cancer survival, and generally indicated that there was poorer survival among GCS with CCD, compared with those without CCD [9–13].

Although much is known about survival rates and complications of therapy for gynecologic cancers, the issue of QOL has been addressed only recently. Gynecologic cancers pose special risks for QOL [14, 15]. Given the challenges and changes that women must face after a diagnosis of gynecologic cancer, QOL is an especially pertinent issue that needs attention. Evaluating and addressing QOL issues have been an important part of the whole package of modern medical care in GCS [16].

The potential QOL deficits associated with GCS who has CCD may be quite large. However, despite the recognition of the link between CCD and GCS, very little has been known about the impact of CCD on QOL in these patients. The objectives of this study were to examine the prevalence of CCD and to estimate the impact of CCD on QOL in the following groups: 1) GCS with CCD compared to GCS without CCD, and 2) GCS with one kind of CCD compared to GCS without it. A better understanding of the effects of CCD on QOL is needed to improve quality of care among GCS.

## Methods

### Recruitment

From April to July 2013, we consecutively contacted GCS in multi-community cancer rehabilitation centers, all of which were affiliated Shanghai Cancer Rehabilitation Club, Shanghai, China- a non-government organization exclusively for cancer survivors. It covered all the 17 districts in Shanghai and has about 13500 cancer survivor members in Shanghai by the end of 2012. This peer support group aims at improving survivors’ QOL. The SCRC regularly offers rehabilitation activities including physical exercise, relaxation training, counseling, psychotherapy and a variety of leisure activities [17].

There were 899 GCS registered in the SCRC by the end of 2012. All members who intended to continue to participate in the SCRC were required to register annually, including the former members and new members. The survey invitations were sent through short text messages and/or phone calls to all 899 GCS. Among all these GCS, 70 were unable to reach possibly because of deaths, migration or refusing to response; 231 declined to participate the survey either because they had no time, or because their health status or literacy ability was too poor. Finally, 598 participated in this survey and formed our final sample, and completed the self-administered questionnaire, which normally took 40–60 minutes. Ethical approval to conduct this study was granted by the Medical Research Ethics Committee of the School of Public Health, Fudan University (Protocol number RB #2013–04–0450). Written informed consent was obtained from each participant.

### Instruments

#### Socio-demographic characteristics

We collected information on age, gender, educational level, Body Mass Index, and other socio-demographic information.

#### Comorbid chronic diseases

Participants were asked to indicate either “yes” or “no” on a list of CCD including hypertension, diabetes mellitus, heart and cardiovascular diseases, respiratory diseases, digestive diseases, musculoskeletal diseases.

#### EORTC QLQ-C30

The EORTC QLQ-C30 (version 3.0) was designed and validated to assess QOL [18]. It is a 30-item patient self-rating questionnaire that can be applied to all cancer patients. The EORTC QLQ-C30 is composed of a global health status/QOL-score, five (multi-item) function scales (physical, role, social, emotional and cognitive functions), three multi-item symptom scales (fatigue, pain, nausea), and five single items (dyspnea, insomnia, appetite loss, constipation, diarrhea). A final item evaluates the perceived economic consequences of the disease. Each item has four response options: (1) “not at all”, (2) “a little”, (3) “quite a bit”, and (4) “very much” – except the two items of the global health-status/quality of life scale which have response options ranging from (1) “very poor” to (7) “excellent”. According to the guidelines provided by the EORTC, all scores of the QLQ-C30 were transformed linearly so that all scales ranged from 0 to 100. A higher score for functional scales indicates a healthier level of functioning, and a higher score for global health status/ QOL indicates a higher QOL. A higher score on the symptom scale and on single items indicates a higher level of symptoms or problems [19].

### Statistical analysis

Standard statistical methods were used to examine potential differences in covariates including chi-squared tests for categorical variables and one-way analysis of variance (ANOVA) for continuous variables followed by Bonferroni post hoc tests. CCD was defined in the statistical analyses as a dichotomous variable (yes/no). Multivariate linear regression models were used to compute regression coefficients (β) and associated 95 % confidence intervals as estimates of the mean difference of QOL scores associated with the presence of absence of CCD, adjusting for potential confounding variables. The following potentially confounding variables were included in all regression models: age (continuous), Body Mass Index(continuous), years since diagnosis(continuous), household income (continuous), education (less than junior high school, junior high school or Junior high school), current marital status (married/living with partner or divorced/widowed/ separated/single), and treatment(surgery, chemotherapy, and/or traditional Chinese medicine). Tests for trend were performed by entering the categorical variables as continuous parameters in the model. Statistical tests were based on a two-tailed probability with a significance level of 0.05. All statistical analyses were performed using SAS.9.3 (SAS Institute, Cary, NC).

## Results

### The characteristics of the study sample

All 899 GCS registered in the SCRC were invited to participate, of whom 598 (66.5 %) responded to the survey. The characteristics of the participants are shown in Table 1. Forty-three percent of the sample was 60 years old or above. Majority of them were married (84.4 %) and had at least one CCD (76.9 %). Nearly 35 % of the participants were overweight or obese. Some statistically significant differences between cancer types were noted.

**Table 1:** Socio-demographic and treatment characteristic of GCS (*N* = 598)

Characteristics	Total		Cervical cancer		Ovarian cancer		Endometrial cancer		*P*
	*N* = 598	%	*N* = 224	%	*N* = 299	%	*N* = 75	%	
Age(year)									0.000
<50	88	14.7	56	25.0	23	7.7	9	12.0	
50-59	251	42.0	94	42.0	131	43.8	26	34.7	
60-69	195	32.6	47	21.0	118	39.5	30	40.0	
≥70	64	10.7	27	12.1	27	9.0	10	13.3	
Body Mass Index									0.044
<18.5	29	4.8	14	6.3	11	3.7	4	5.3	
18.5-24	360	60.2	137	61.2	190	63.5	33	44.0	
25-29	185	30.9	66	29.5	87	29.1	32	42.7	
≥30	24	4.0	7	3.1	11	3.7	6	8.0	
Years since diagnosis									0.001
<2	68	11.4	34	15.2	29	9.7	5	6.7	
2-5	175	29.3	82	36.6	72	24.1	21	28.0	
≥6	355	59.4	108	48.2	198	66.2	49	65.3	
Marital status									0.433
Married/with partner	505	84.4	189	84.4	249	83.3	67	89.3	
Divorced/widowed/ separated/ single	93	15.6	35	15.6	50	16.7	8	10.7	
Education									0.015
Less than junior high school	61	10.2	33	14.7	26	8.7	2	2.7	
Junior high school	243	40.6	94	42.0	117	39.1	32	42.7	
More that junior high school	294	49.2	97	43.3	156	52.2	41	54.7	
Household income(yen/month)									0.000
<2000	187	31.3	98	43.8	74	24.7	15	20.0	
2000-4000	322	53.8	94	42.0	180	60.2	48	64.0	
>4000	89	14.9	32	14.3	45	15.1	12	16.0	
Treatment									
Surgery	532	89.0	197	87.9	264	88.3	71	94.7	0.240
Radiotherapy	179	29.9	99	44.2	55	18.4	25	33.3	0.000
Chemotherapy	435	72.7	137	61.2	254	84.9	44	58.7	0.000
Traditional Chinese Medicine	293	49.0	80	35.7	145	48.5	39	52.0	0.005
No. of comorbid chronic diseases									0.004
0	138	23.1	65	29.0	61	20.4	12	16.0	
1	138	23.1	59	26.3	68	22.7	11	14.7	
2	119	19.9	40	17.9	64	21.4	15	20.0	
≥3	203	33.9	60	26.8	106	35.5	37	49.3	
Comorbid chronic disease									
Hypertension	184	30.8	60	26.8	95	31.8	29	38.7	0.135
Diabetes	106	17.7	25	11.2	59	19.7	22	29.3	0.001
Heart and Cardiovascular	147	24.6	49	21.9	75	25.1	23	30.7	0.298
Respiratory diseases	49	8.2	17	7.6	22	7.4	10	13.3	0.221
Digestive diseases	297	49. 7	104	46.4	149	49.8	44	58.7	0.185
musculoskeletal diseases	191	31.9	68	30.4	97	32.4	26	34.7	0.760

In terms of the types of CCD, the proportion of GCS who suffered from digestive diseases (49.7 %) was the highest. Other CCD are listed according to their prevalence as follows: hypertension (30.8 %), diabetes mellitus (17.7 %), heart and cardiovascular diseases (24.6 %), respiratory diseases (8.2 %), digestive diseases (49.7 %), musculoskeletal diseases (31.9 %). Meanwhile, the highest overall prevalence of all CCD was found in endometrial cancer survivors. The prevalence of diabetes was significantly different between cancer types.

### The influence of comorbid chronic diseases on EORTC QLQ-C30 scores

The influences of CCD on EORTC QLQ-C30 scores are presented in Table 2. After adjusting for the influence of the socio-demographic variables, subjects with self-reported CCD generally reported lower scores for most EORTC QLQ-C30 scales when compared to subjects without these CCD, indicating poorer QOL. The influences of heart and cardiovascular diseases, respiratory diseases and musculoskeletal illnesses on EORTC QLQ-C30 scores were of a similar magnitude and were larger than the influence of hypertension, diabetes or digestive diseases.

**Table 2:** Quality of life for subjects with and without comorbid chronic diseases after adjusting for the influence of the socio-demographic variables

	CCD, *n* = 460	HPT, *n* = 184	DM, *n* = 106	HD, *n* = 147	RD, *n* = 49	DD, *n* = 297	SD, *n* = 191
	(no CCD, n = 138)	(no HPT, *n* = 414)	(no DM, *n* = 492)	(no HD, *n* = 451)	(no RD, *n* = 549)	(no DD, *n* = 301)	(no SD, *n* = 407 )
EORTC QLQC-30							
PF	−2.50(83.46)	−5.43(83.09) **	−3.87(82.20)	−6.95(82.95) **	−7.79(82.07) *	−1.46(82.28)	−5.58(83.31) **
RF	−3.66(92.49)	−4.73(90.99) *	−6.44(90.77) *	−8.21(91.32) **	−14.94(90.71) ***	−1.50(90.41)	−4.72(91.13) *
CF	−5.81(87.86) *	−3.24(84.22) **	−4.28(84.05)	−5.94(84.51) *	−9.76(83.99) **	−4.88(85.93) **	−8.99(86.20) ***
EF	−5.71(81.92) *	−3.93(78.56) **	−4.88(78.30) *	−5.70(78.60) *	−7.00(77.93) *	−5.02(80.15) **	−6.60(79.56) **
SF	−5.71(80.59)	−5.69(77.75) *	−1.90(76.42)	−7.61(77.67) *	−15.34(77.22) **	−4.81(78.70) *	−8.67(78.90) **
QL	−7.22(65.93) *	−4.37(61.50) **	−5.69(61.26)	−9.25(62.16) **	−13.19(61.20) **	−4.00(62.40)	−11.26(63.89) ***
FA	8.82(23.22) **	5.00(28.72) *	2.84(29.68)	10.09(28.07) ***	9.42(29.49) *	8.55(25.52) ***	7.96(27.60) **
NV	0.39(3.20)	1.60(3.04)	2.79(3.00) *	3.29(2.82) **	3.48(3.25)	−0.58(3.83)	1.29(3.09)
PA	3.22(15.29)	6.94(15.79) **	2.83(17.32)	9.40(15.86) ***	13.43(16.83) ***	1.04(17.27)	12.85(13.64) ***
DY	6.90(9.47) **	1.79(14.40)	1.93(14.58)	10.63(12.69) ***	21.24(13.34) ***	4.28(12.59) *	4.38(13.50) *
SL	7.99(14.90) *	2.43(20.51)	3.46(20.59)	10.13(19.09) **	16.22(20.01) ***	5.92(17.99) *	9.11(18.25)
AP	3.89(6.36)	2.83(8.61)	2.69(8.95)	3.76(8.65)	9.93(8.70) **	−0.03(9.46)	6.95(7.17) **
CO	6.05(7.88) *	0.29(12.59)	4.86(11.78)	7.71(11.05) **	12.11(11.76) **	3.29(10.87)	3.70(11.46) **
DI	4.39(5.08)	0.06(8.54)	0.48(8.47)	1.87(8.16)	11.55(7.69) ***	1.53(7.72)	2.95(7.59)
FI	4.82(30.89)	5.34(33.13)	5.50(33.69)	9.67(32.67) *	13.54(33.69) *	4.72(32.12)	2.71(33.82)

1.Quality of life questionnaire abbreviation: Physical Functioning PF;Role Functioning, RF;Cognitive Functioning, CF;Emotional Functioning, EF;Social Functioning, SF;Global Health,QL; Fatigue, FA; Nausea and vomiting, NV; Pain, PA; Dyspnoea, DY; Insomnia, SL; Appetite loss, AP; Constipation, CO; Diarrhoea, DI; financial difficultis, FI. Social Functioning, SF

2. Comorbid chronic diseases abbreviation: Comorbid chronic diseases, CCD; hypertension, HPT;diabetes mellitus, DM; heart and cardiovascular diseases, HCD; respiratory diseases, RD; digestive diseases, DD; musculoskeletal diseases, MD

3. The difference in mean score of quality of life between GCS with and without CCD (mean score of quality of life among GCS without CCD)

4. Multiple linear regression, adjusted for influence of gender, age, BMI, education, household income, time after diagnosis, treatment.

5. * *P* <0.05, ** *P* <0.01, *** *P* <0.001

## Discussion

Based on our data, the prevalence of the CCD including hypertension, diabetes mellitus, heart and cardiovascular diseases, respiratory diseases, digestive diseases, musculoskeletal diseases was quite high among GCS. Seventy seven percent of GCS had at least one of the CCD considered in this study. Furthermore, the prevalence of multiple CCD was significant in endometrial cancer survivors, with nearly half of them suffering from two or more CCD. This study indicated that QOL scores of GCS with CCD were lower than those without reporting the corresponding CCD.

Previous studies have shown that patient demographic characteristics such as age, sex, marital status, and education are important factors associated with cancer QOL [20, 21], and the main purpose of this study was to understand the impact of CCD on QOL; therefore, multiple linear regression models were used to control for the effects of socio-demographic characteristic.

Cancer survivors with CCD have clinical and health care needs that may differentiate them from persons without CCD. Evidence indicates that persons with one CCD are more likely to have other CCD [22, 23]. The GCS in our study reported that more than half of them have more than one CCD. Moreover, cancer survivors with severe CCD may have more rapid declines in health status and a greater likelihood of disability and mortality [24–26]. In China, the management of chronic diseases and cancer was often fragmented, for the management of cancer was more focused on cancer recurrence and metastasis. The significant impact of CCD on the health and QOL of cancer patients, however, was easily overlooked.

Our study indicated that the CCD impacts the QOL of GCS. Added to known cancer-specific vulnerabilities, the increased risk of CCD makes the need for comprehensive care programs for GCS even more urgent. Given the challenges and changes that women must face after a diagnosis of gynecologic cancer, QOL is an especially pertinent issue that we ought to focus on. Understanding the long-term physical and psychosocial effects of cancer and CCD can help gynecologic patients and their families better deal with potential health risks associated with being a cancer survivor. CCD prevention and control will help improve the QOL and the overall health of GCS.

Obesity is a serious health problem which has significant impact on the incidence and treatment of the gynecologic cancers, especially for endometrial cancer [27, 28]. Obese women with cancers have decreased survival rate which may be due to disease-specific reasons [29], the result of comorbid illnesses [30], or response to treatment [31]. In our study, nearly 35 % of subjects were overweight and obese. In a nationally representative sample of 15,540 Chinese middle-aged and elderly adults, the prevalence of overweight and obesity were 36.9 % in men and 31.1 % in women, respectively [32]. Obviously, the prevalence of overweight and obese in GCS was very high. The China National Diabetes and Metabolic Disorders Study, conducted from June 2007 to May 2008, revealed that 8.8 % of all women aged 20 years or older had diabetes, 0.51 % had coronary heart disease, 0.60 % had had a stroke and 1.10 % had cardiovascular disease [33]. Comparing with the general Chinese population, the prevalence of CCD in our cohort was also very high. Meanwhile, the highest overall prevalence of all CCD was found in endometrial cancer survivors.

High calorie intake and little physical activity are associated with obese and often result in many other comorbidities e.g., diabetes mellitus, hypertension, dyslipidemia, coronary heart disease, gallbladder disease, arthritis and constipation [34]. The association between the CCD and QOL among GCS warrants changes in the lifestyle for patients and also challenges old paradigms that oncology’s work is done after treatment. Collaborative chronic disease management models may be particularly appropriate in this regard, which emphasizes continuity of long-term care and a relationship between patient and provider in which the patient is empowered and takes an active role in their ongoing care such as making lifestyle changes [35, 36].

Our study has several limitations that merit discussion. Firstly, all the subjects were recruited from the Cancer Rehabilitation Club. The most common reason that GCS attended the SCRC perhaps was that they were more unwell and feel a need for support and rehabilitation. Possibly, the QOL of our sample was worse compared with the overall population of Chinese GCS. It was also necessary that comparing the difference of QOL between the more broad population of Chinese GCS and other types of cancer survivors, moreover, and women of the general population who have CCD for future studies. Second, information on cancer staging was not collected in this study. Examining the association between cancer staging and QOL will be the focus of our future study. Finally, the validity of self-reported CCD could be questionable [37], however, in our study, we made it clear to the respondents that the CCD must be a clinical diagnosis. These self-report CCD items could be a viable tool to collect comorbidity data [38].

## Conclusions

There exists an association between CCD and QOL among Chinese GCS, and subjects with CCD generally reported lower QOL scores. These findings warrant the comprehensive cares for GCS. A multidisciplinary team approach and a variety of delivery systems are needed to address the medical, psychosocial, and lifestyle components of gynecological cancer survivorship care.

### Availability of data and materials

Not applicable.
